# Diet Quality, Nutrient Intake, and Body Fat Percentage in Women with Infertility and Normal Body Mass Index

**DOI:** 10.3390/nu18111775

**Published:** 2026-05-31

**Authors:** Adriana Szulińska, Barbara Grzechocińska, Monika Grymowicz, Piotr Sobieraj, Agnieszka Bzikowska-Jura

**Affiliations:** 1 Department of Clinical Dietetics, Medical University of Warsaw, 00-575 Warszawa, Poland; abzikowska@wum.edu.pl; 2 1st Department and Clinic of Obstetrics and Gynecology, Medical University of Warsaw, 02-015 Warszawa, Poland; barbara.grzechocinska@wum.edu.pl; 3 Department of Gynecological Endocrinology, Medical University of Warsaw, 2 Karowa Street, 00-315 Warsaw, Poland; monika.grymowicz@wum.edu.pl; 4 Department of Internal Medicine, Hypertension and Vascular Diseases, Faculty of Medicine, Medical University of Warsaw, Banacha Str. 1a, 02-097 Warsaw, Poland; piotr.sobieraj@wum.edu.pl

**Keywords:** infertility, normal-weight obesity, body fat percentage, diet quality, protein intake, sucrose, female reproductive health

## Abstract

Background: Infertility is a major public health concern worldwide. While excess body weight is a well-established risk factor for impaired fertility, increasing evidence indicates that elevated body fat content may also occur in women with normal body mass index, a phenotype described as normal-weight obesity. This study aimed to assess associations between diet quality, intake of selected nutrients, and body fat content among women attending an infertility clinic with body mass index (BMI) within the normal range. Methods: This cross-sectional study included 45 women with infertility and normal BMI (18.5–24.99 kg/m^2^). Dietary intake was assessed using 3-day food diaries and a food frequency questionnaire. Diet quality was evaluated using the pro-healthy diet index, non-healthy diet index, and diet quality index. Body composition was measured by bioelectrical impedance analysis. Participants were divided according to median body fat percentage, <24.9% vs. ≥24.9%. Results: Participants’ body fat percentage ranged from 12.2% to 34.3%, and in the case of 6 women, the body fat percentage (>30%) indicated normal weight obesity. No differences were observed in pHDI, nHDI, or DQI scores between groups after correction for multiple comparisons. Inadequate iron intake was reported in all women. Conclusions: Among women with infertility and normal BMI, body fat percentage varied widely. No associations were observed between dietary variables and body composition parameters.

## 1. Introduction

Infertility is defined as the failure to achieve a clinical pregnancy after 12 months of regular unprotected sexual intercourse. This condition affects up to 186 million individuals worldwide, predominantly in developed countries, highlighting its significance as a major public health concern. Among non-medical factors that adversely affect fertility, female age plays a crucial role; however, lifestyle-related factors are also known to be of substantial importance [1]. These factors may exert long-term effects on women’s health and contribute to a wide range of physiological dysfunctions, including impaired fertility [2].

Poor dietary habits and obesity are among the key modifiable lifestyle factors associated with impaired fertility. Excess body weight has been associated with ovulatory disorders and reduced effectiveness of assisted reproductive technologies (ART). Importantly, a woman’s diet influences not only the likelihood of successful conception but also multiple aspects of normal fetal development. Maternal obesity is linked not only to an increased risk of perinatal complications but also to a higher incidence of congenital anomalies [2]. On the other hand, it is well established that individuals with a normal body mass index (BMI) may still present with excessive body fat mass, which is associated with increased cardiometabolic risk. This observation suggests that BMI assessment without body composition analysis may be insufficient for accurate evaluation of health risk [3]. This condition, referred to as “normal-weight obesity,” often remains undiagnosed due to the lack of routine body composition measurements in clinical practice [4].

In the context of fertility, higher body fat content has been associated with a longer time to pregnancy [5]. Moreover, women with normal weight and elevated body fat percentage may experience poorer outcomes of in vitro fertilization, including a lower number of antral follicles, high-quality embryos, retrieved oocytes, and fertilized oocytes [6]. At the same time, growing evidence confirms an association between dietary indices and female fertility [7,8].

The aim of this study was to assess the relationship between diet quality, intake of selected nutrients, and body fat content among women attending an infertility clinic who had normal BMI. In particular, the study sought to compare diet quality indices and selected nutrient intake according to body fat percentage, and to determine whether women with higher body fat content, despite normal BMI, exhibit lower diet quality or different dietary patterns compared with women with lower body fat content. Identification of these associations may support improved diagnostics and nutritional counseling in women with infertility.

## 2. Materials and Methods

### 2.1. Study Design

This study was designed as a cross-sectional observational study. The study group included female patients attending infertility clinics at the University Center for Women’s and Newborn Health and the Princess Anna Mazowiecka Clinical Hospital in Warsaw (Poland) between 2022 and 2025. Information about the study was provided to eligible patients in person by the principal investigator (A.S.) and a physician working at the infertility clinic (M.G., B.G.). All participants received detailed information about the study procedures and provided written informed consent prior to participation.

The inclusion criteria were as follows: infertility defined as failure to achieve pregnancy after 12 months of regular attempts, age over 18 years, no history of pregnancy, and BMI within the normal range (18.5–24.99 kg/m^2^). Women were excluded from the study if conditions directly affecting fertility were diagnosed, including tubal obstruction, a history of surgical procedures involving the reproductive organs, as well as in the presence of hormonal disturbances or when infertility in the couple was attributed to male-related factors.

### 2.2. Assessment of Dietary Intake and Diet Quality

To assess nutrient intake, each participant was asked to complete a 3-day food diary. The collected dietary records were subsequently reviewed, clarified, and validated during a structured dietary interview conducted by a dietitian (A.S.). The data from the food diaries were entered into the Dieta 6.0 software to calculate the average daily intake of energy and nutrients.

Additionally, all participants completed a lifestyle questionnaire that included a food frequency questionnaire (FFQ). To calculate diet quality indices, 10 food groups with potentially beneficial health effects and 14 food groups with potentially adverse health effects were included (Table 1).

The frequency of food consumption was assessed using a six-point scale (1–6), which was subsequently converted into daily frequency coefficients (times/day), as presented in Table 2.

To evaluate diet quality, the pro-healthy diet index (pHDI-10; range: 0–20) was calculated by summing the daily frequency coefficients for the 10 pro-healthy food groups. The non-healthy diet index (nHDI-14; range: 0–28) was calculated analogously by summing the daily frequency coefficients for the 14 non-healthy food groups.

For standardization purposes, both indices were converted into point-based scores ranging from 0 to 100. The pHDI-10 score was calculated by multiplying the sum of the daily frequency coefficients for the 10 pro-healthy food groups by 100/20, whereas the nHDI-14 score was calculated by multiplying the sum of the daily frequency coefficients for the 14 non-healthy food groups by 100/28. Values of 0–33, 34–66, and 67–100 represented low, moderate, and high intensity of pro-healthy or non-healthy dietary characteristics, respectively.

Subsequently, based on the pHDI-10 and nHDI-14 indices, the overall diet quality index (DQI; range: −100 to 100) was calculated according to the following formula:

DQI = (100/20) × Σ frequency of consumption of 10 pro-healthy food groups (times/day) + (−100/28) × Σ frequency of consumption of 14 non-healthy food groups (times/day),

where values from −100 to −26 indicate a high intensity of unhealthy dietary characteristics, values from −25 to 25 indicate a low intensity of both unhealthy and pro-healthy characteristics, and values from 26 to 100 indicate a high intensity of pro-healthy dietary characteristics. The methodology was based on the validated KomPAN^®^ questionnaire [9].

### 2.3. Anthropometric Measurements and Body Composition Analysis

For each participant, body height (measured to the nearest 1 cm) and body weight (measured to the nearest 0.1 kg) were recorded, and BMI was calculated using the following formula: BMI = body weight (kg)/height (m)^2^. Waist circumference was measured at the midpoint between the lower margin of the last rib and the iliac crest, and hip circumference at the widest point at the level of the buttocks. These measurements were used to calculate the waist-to-hip ratio (WHR) according to the formula: WHR = waist circumference/hip circumference.

After excluding contraindications (pregnancy, epilepsy, metal implants in the body, e.g., pacemaker, endoprosthesis and presence of permanently installed electronic devices or stimulators), body composition was assessed using bioelectrical impedance analysis. Measurements were performed using the TANITA BC-1000 device (Tanita, Tokyo, Japan). The device estimates body composition parameters using proprietary prediction algorithms based on impedance measurements. Prior to the assessment, all participants received standardized instructions regarding preparation for body composition analysis to ensure uniform measurement conditions. These recommendations included a last meal ≥ 3 h before the test, no fluids 1 h before (small amounts of water allowed) with normal hydration in the preceding 24 h, an empty bladder, avoiding intense physical activity for 12 h, no caffeine for 3 h, no alcohol for 24 h, and no foot cream on the day of measurement.

Based on the obtained results, participants were divided into two groups according to total body fat percentage, using the median value as the cut-off point. This data-driven grouping approach was exploratory and was not intended to represent a clinically validated threshold for normal-weight obesity. Additionally, this strategy allowed for the establishment of comparable group sizes and enabled comparison of women with relatively lower versus higher body fat percentage within a homogenous population of women with normal BMI.

### 2.4. Statistical Analysis

In the statistical analysis, variables with a normal distribution were presented as mean ± standard deviation, while continuous variables not meeting the assumption of normality were described using the median and interquartile range. Normality of distribution was assessed using the Shapiro–Wilk test. Comparisons between groups were performed using Student’s *t*-test for normally distributed variables and the Mann–Whitney U test for variables not meeting the assumption of normality. Cohen D and Wilcoxon r indices were used to report effect size. Categorical variables were compared using the chi-square test.

Categorical variables were expressed as counts and corresponding percentages. Associations between variables were evaluated using correlation analysis, applying Pearson’s correlation coefficient for normally distributed variables and Spearman’s rank correlation coefficient when the assumption of normality was not met. All *p*-values (except for the comparison of pHDI, nHDI and DQI between groups and comparison of food consumption frequencies) were reported as corrected using the Benjamini–Hochberg procedure. *p*-value < 0.05 was considered statistically significant. Food consumption frequency comparisons are only exploratory and should be interpreted with caution.

Penalized regression analysis was additionally performed using the least absolute shrinkage and selection operator (LASSO) method to identify the most relevant predictors of dietary indices and nutrient intake parameters using linear regression. The optimal regularization parameter (λ) was selected using 10-fold cross-validation based on the minimum cross-validated mean squared error. The following variables were entered as candidate predictors: age, fat mass (%), body mass index (BMI), body mass, and waist-to-hip ratio (WHR). Separate LASSO regression models were constructed for each dependent variable, including pHDI, nHDI, DQI, dietary energy intake, protein intake, protein (g/kg body weight), energy from protein (%), fat intake (g), energy from fat (%), carbohydrates intake (g), and energy from carbohydrates (%). Model tuning was performed using cross-validation to determine the optimal value of the regularization parameter (λ) minimizing prediction error. Variables with coefficients shrunk to zero were considered not to contribute independently to the model. Regression coefficients obtained from the final penalized models were reported as measures of the direction and magnitude of associations.

All statistical analyses were performed using R (version 4.5.3; R Foundation for Statistical Computing, Vienna, Austria).

## 3. Results

### 3.1. Characteristics of the Study Group

A total of 45 women aged 33.4 ± 3.3 years were enrolled. BMI was 21.1 ± 2.1 kg/m^2^ and body fat percentage among the study participants varied between 12.2% and 34.3% (median 24.9; IQR 20.1–26.8), with six individuals in Group II exhibiting a body fat level exceeding 30%. The median body fat percentage, used as the cut-off point for defining the comparison groups, was 24.9%. Group I included women with a body fat percentage < 24.9%, whereas Group II included women with a body fat percentage ≥ 24.9%. Despite the inclusion criterion requiring a BMI within the normal range, the two groups differed significantly in terms of BMI values (19.5 ± 1.3 vs. 22.6 ± 1.4 kg/m^2^; q < 0.001; Cohen’s d = 2.336). No significant differences in age were observed between the groups. A detailed characteristic of the study sample, described in groups by body fat percentage, is presented in Table 3.

### 3.2. Dietary Intake

No differences in nutrient intake remained significant following adjustment for multiple comparisons. Energy intake and the consumption of selected nutrients are presented in Table 4.

### 3.3. Adequacy of Nutrient Intake

In the study group, five women (two in Group I and three in Group II) had protein intake per kilogram of body weight below recommended values [10]. In contrast, in 13 participants, the percentage of energy derived from fat exceeded the maximum recommended level.

Notably, the quality of fat intake was suboptimal, as 73.3% of the women reported intake of EPA + DHA below the recommended level of 250 mg/day. Intake of saturated fatty acids exceeding recommended limits was observed in 62.2% of participants. The most pronounced irregularities in macronutrient intake concerned simple sugars, with 82.2% of women exceeding recommended intake levels.

Regarding mineral intake, all study participants consumed iron in amounts below the recommended intake [10]. A high proportion of women (86.7%) also failed to meet calcium intake recommendations [10]. A detailed overview of nutrient intake adequacy in relation to the Polish Nutritional Standards is presented in Table 5.

### 3.4. Frequency of Consumption of Food Products

**Figure 1:**
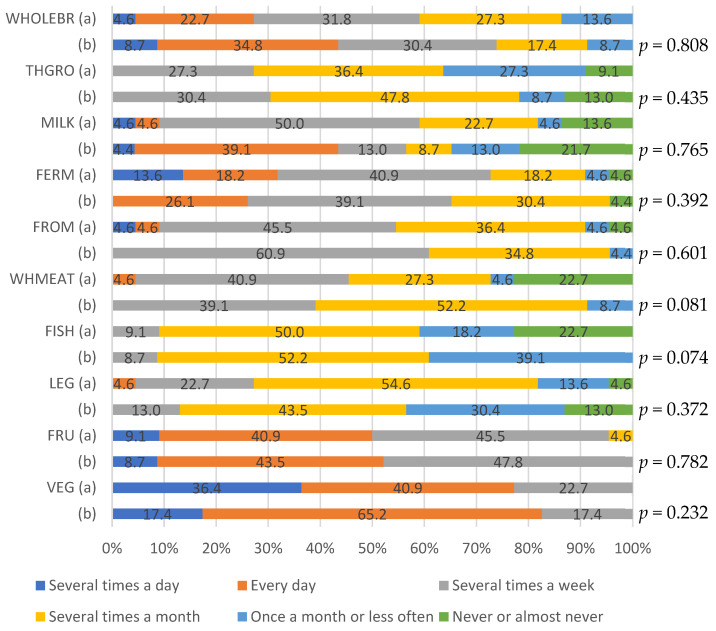
Percentage of responses for health-promoting products in Group I (a) and Group II (b). Differences in the distribution of responses between groups were assessed using the chi-square test with exact *p*-values. WHOLEBR—Wholemeal bread, THGRO—Buckwheat groats, oat flakes, whole-grain pasta or other coarse groats, MILK—Milk (including flavored milk, cocoa, coffee with milk), FERM—Fermented milk beverages, e.g., yogurts and kefirs (natural or flavored), FROM—Cottage cheeses (including homogenized cheeses and cottage-cheese desserts), WHMEAT—Dishes prepared from so-called white meat, e.g., chicken, turkey, rabbit, FISH—Fish, LEG—Dishes prepared from legume seeds, e.g., beans, peas, soybeans, lentils, FRU—Fruits, VEG—Vegetables.

Figure 1 illustrates the distribution of responses regarding the frequency of consumption of food products with potentially beneficial health effects in Group I and II, respectively. Only 27.3% of participants in Group I and 43.5% in Group II reported consuming whole-grain bread at least once daily. All participants declared consuming coarse groats only a few times per week or less frequently. Fish consumption in accordance with dietary recommendations (at least several times per week) [10] was reported by only 9.1% of participants in Group I and 8.7% in Group II. Daily consumption of vegetables and fruits was also not reported by all participants.

**Figure 2:**
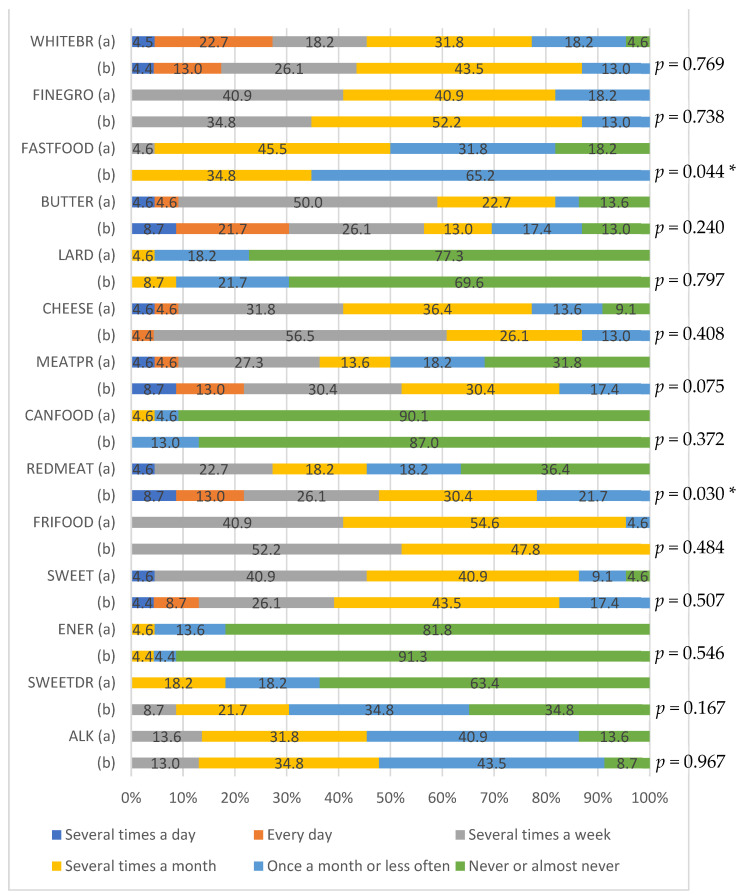
Percentage of responses for non-health-promoting products in Group I (a) and Group II (b). Differences in the distribution of responses between groups were assessed using the chi-square test with exact *p*-values. WHITEBR—White bread and bakery products (e.g., wheat bread, toasted bread, white bread rolls), FINEGRO—White rice, white pasta, fine groats (e.g., semolina, couscous), FASTFOOD—Fast food (e.g., French fries, hamburgers, pizza, hot dogs, casseroles), BUTTER—Butter (for bread spread or as a side dish for frying, baking, etc.), LARD—Lard (for bread spread or dishes. for frying, baking, etc.), CHEESE—Cheeses (including processed and blue cheeses), MEATPR—Processed meats (sausages, cold cuts, pâtés), CANFOOD—Canned meats, REDMEAT—Red meat (e.g., pork, beef, veal, mutton, lamb, game), FRIFOOD—Fried foods (e.g., meat or flour-based dishes), SWEET—Sweets, such as candies, cookies, cakes, chocolate bars, muesli bars, and other confectionery products, ENER—Energy drinks, SWEETDR—Sweetened carbonated or non-carbonated beverages, ALK—Alcoholic drinks; * statistically significant (*p* < 0.05).

Figure 2 presents analogous distributions for food products with potentially adverse health effects. For non-health-promoting products, the responses “never or almost never” and “once a month or less often” were reported more frequently than for health-promoting products. Daily consumption of these products was rare but occurred more often in Group II. For products such as red meat, processed meat, and fried foods, participants in Group II more frequently reported consumption several times per week or more often. Women in Group II reported more frequent consumption of red meat (*p* = 0.030) and fast-food products (*p* = 0.044) in analyses not adjusted for multiple comparisons.

### 3.5. Diet Quality Indices

No differences were observed between the groups in the pro-healthy diet index (pHDI), non-healthy diet index (nHDI), or the overall diet quality index (DQI). Detailed values of diet quality indices for the total study sample and stratified by body fat percentage are presented in Table 6.

### 3.6. Correlation Analysis

As part of the study, correlation analyses were performed between anthropometric measurements, nutrient intake, and diet quality indices. After applying the Benjamini–Hochberg false discovery rate correction, we did not observe any associations between nutritional data and body composition and anthropometric parameters. Detailed results of correlation analysis are presented in Supplementary Table S1.

### 3.7. Multivariable Models

In the LASSO regression analysis, five potential predictors were included: fat (%), BMI, age, body weight, and WHR. For most dependent variables, the LASSO model with the optimal regularization parameter (λ) eliminated all predictors, leaving only the intercept term. This was observed for pHDI, DQI, protein intake, energy intake from protein (%), energy intake from fat (%), and energy intake from carbohydrates (%).

For nHDI, dietary energy intake, and carbohydrates, the final models retained single coefficients with values close to zero (for age or fat (%), respectively), indicating a lack of meaningful predictive contribution after LASSO regularization.

Only for protein per kg of body mass did the model retain two predictors, fat (%) and BMI, both showing negative regression coefficients (β = −0.012 for fat (%) and β = −0.030 for BMI). In contrast, the model for fat intake retained BMI (β = −3.70), age (β = −0.69), and body weight (β = 0.66).

Overall, the LASSO analysis suggests that among the analysed anthropometric and clinical variables, only selected parameters demonstrated limited predictive value for certain nutritional outcomes, whereas for most analysed endpoints no stable associations were identified after regularization.

## 4. Discussion

The present study assessed the relationship between diet quality, selected nutrient intake, and body fat content among women attending an infertility clinic who had a normal BMI. The findings indicate that despite normal BMI, body fat percentage varied considerably, and six participants exhibited body fat percentage > 30%, consistent with the concept of normal-weight obesity [11]. This supports previous evidence suggesting that BMI alone may be insufficient for identifying unfavorable body composition profiles in women of reproductive age [12].

In the context of infertility, excess adiposity despite normal BMI may negatively affect ovarian function, time to pregnancy, and outcomes of assisted reproductive technologies [5,6]. The present findings support the potential value of body composition assessment in routine infertility diagnostics, rather than relying exclusively on BMI.

Studies evaluating the relationship between diet quality indices and BMI have yielded inconsistent findings [13,14,15]. Some studies suggest an inverse association, while others do not confirm such a relationship [14] or report it only in selected subgroups [15]. Evidence also indicates that diet quality indices may be associated with a metabolically unfavorable phenotype in individuals with normal BMI [16,17]. However, data on their relationship with fertility parameters remain scarce. For example, higher DQI scores have been associated with a lower risk of diminished ovarian reserve and with more favorable ovarian reserve parameters among women with diminished ovarian reserve [7].

In the present study, no significant differences were observed between groups in the dietary quality indices (pHDI, nHDI, and DQI), despite differences in selected nutrients and food product consumption frequencies in descriptive analyses. Importantly, most observed associations did not retain significance following adjustment for multiple comparisons, indicating that the findings should be interpreted cautiously and require confirmation in larger studies. Additionally, the indices used in this study were based on broad food group classifications and frequency-based assessment, which may have further limited their discriminatory ability.

Overall, the LASSO procedure resulted in sparse models, suggesting that most of the examined variables did not demonstrate substantial independent predictive utility for the analyzed dietary outcomes; however, fat mass (%) retained importance in some of the models, suggesting the relationship between dietary indices and fat mass (%). Previous studies suggest that adequate protein intake may play a role in metabolic homeostasis and hormonal balance in women of reproductive age [18]. In this study, protein intake was expressed relative to body weight, which may introduce partial mathematical dependency between variables related to body size and body composition rather than reflecting physiological relationships alone. Moreover, protein intake expressed per kilogram of body weight was strongly correlated with total energy intake, which may have further complicated interpretation of the observed results. Importantly, none of the associations between protein intake and anthropometric parameters remained statistically significant after Benjamini–Hochberg correction for multiple comparisons.

Previous studies suggest that excessive sucrose intake may be associated with insulin resistance and low-grade inflammation, which have been linked to impaired ovarian function and reproductive health [2]. Meanwhile, 80% of the participants exceeded the recommended upper limit for sugar intake relative to total daily energy intake.

Despite the lack of differences in diet quality indices, some differences were observed in the frequency of consumption of specific food groups. Women with higher body fat percentage reported more frequent consumption of red meat and fast-food products. This exploratory observation may be of interest, as previous studies suggested that higher red meat consumption has been linked to ovulatory infertility, particularly in the case of processed red meat, and to conditions impairing fertility, such as endometriosis [19,20,21,22]. Importantly, the compared groups differed not only in body fat percentage, but also in BMI, body weight, and selected anthropometric parameters related to central adiposity. Consequently, it cannot be excluded that some of the observed differences were influenced by overall body size and adiposity-related characteristics rather than body fat percentage alone.

Another important aspect of the present study is the widespread inadequacy of key nutrient intake observed across the study population. All participants failed to meet the recommended intake for iron, and the majority did not reach recommended levels for calcium, dietary fiber, and long-chain omega-3 fatty acids (EPA and DHA). Iron deficiency is of particular concern in women of reproductive age, as it may impair ovulatory function and adversely affect pregnancy outcomes [10]. In Europe, it affects 10–32% of women in this group [23], and higher iron intake has been associated with a lower risk of infertility [24]. In pregnant women, inadequate iron intake and deficiency remain common and are linked to adverse pregnancy outcomes and impaired fetal development [25,26]. Inadequate calcium intake may also affect hormonal regulation and long-term health outcomes [27,28]. The high prevalence of insufficient omega-3 fatty acid intake observed in this study is also noteworthy. Previous studies have reported that EPA and DHA may exert anti-inflammatory effects and may be associated with improved reproductive parameters, including oocyte quality and implantation potential [2,8]. Higher omega-3 fatty acid intake has been associated with improved fertility outcomes, both from diet and supplementation [29,30]. Accordingly, particular attention should be drawn to the fact that only 20% of the women studied consumed fish, an important source of omega-3 fatty acids, at least several times per week. Taken together, the findings of this study do not support clear associations between dietary variables and body composition parameters after correction for multiple comparisons; however, the observed dietary patterns may warrant further investigation in larger studies of women with infertility and normal BMI.

These findings suggest that assessment of specific dietary components and body composition may provide additional insight beyond global dietary quality indices in women with infertility and normal BMI. Scientific evidence consistently confirms that an appropriate dietary pattern characterized by high consumption of vegetables, fruits, legumes, and fish, along with low intake of processed meat and sugar, is associated with increased chances of becoming pregnant [31]. In practice, however, nutritional consultation for such patients is rarely proposed and, if it is, usually only in cases of overweight or diet-related diseases.

The strengths of this study include comprehensive dietary assessment using both 3-day food diaries and FFQ, as well as objective evaluation of body composition using bioelectrical impedance analysis. However, it should be acknowledged that body composition was assessed using bioelectrical impedance analysis rather than a reference method such as DXA. Although BIA is widely used in clinical practice and research due to its practicality and non-invasive nature, its accuracy may be influenced by factors such as hydration status and device-specific prediction algorithms. Additionally, the observational cross-sectional design and relatively small sample size limit the generalizability and statistical robustness of the findings. Due to the selective inclusion criteria, the study population represented a relatively specific subgroup of women with infertility and normal BMI, which limited recruitment possibilities. Consequently, statistical power may have been limited, particularly in the context of multiple comparisons and correlation analyses. Another limitation is the use of a median-based cut-off to define comparison groups. Although body fat percentage >30% is commonly used as a reference criterion for normal-weight obesity in women, only six participants in the present study exceeded this threshold, which limited the possibility of meaningful statistical comparison. Therefore, a data-driven approach was considered more appropriate for exploratory analyses in this relatively small sample. Dietary intake was also self-reported, which may be subject to recall and reporting bias. Another limitation of this study is the lack of detailed assessment of potential confounding factors such as hormonal profile, insulin resistance, and physical activity. Physical activity was not assessed using a standardized tool, and perceived stress was measured using a simple, non-validated scale, which limited its use in the analysis. Although major hormonal disorders were part of the exclusion criteria, more subtle metabolic or hormonal variations could not be accounted for.

Additionally, the study population represented a selected subgroup of women with idiopathic infertility attending an infertility clinic and may not reflect the full spectrum of infertility patients. However, this approach allowed for greater homogeneity of the study population and reduced the potential influence of clinical conditions that could independently affect dietary behavior, body composition, or motivation toward healthy eating.

## 5. Conclusions

In this exploratory study of women with infertility and normal BMI, body fat percentage varied considerably. No associations between body fat percentage and overall diet quality or selected nutrient intake remained after correction for multiple comparisons. Although selected dietary characteristics differed between groups in additional analyses, these observations should be interpreted cautiously. These findings also indicate that, in this sample, dietary factors did not clearly explain differences in body fat percentage. Further studies in larger populations are needed to verify these observations and to better assess the potential role of body composition in women with infertility and normal BMI. 

## Figures and Tables

**Table 1:** Classification of food groups used for the calculation of diet quality indices [9].

Pro-Healthy Food Groups	Non-Healthy Food Groups
Wholegrain bread and bakery products Buckwheat groats, oatmeal, wholegrain pasta, or other coarse-grained cereals Milk (including flavored milk, cocoa, coffee with milk) Fermented milk beverages (e.g., yogurts, kefir; natural or flavored) Fresh curd cheese products (including cottage cheese, homogenized cheese, curd-based desserts) Dishes prepared from so-called white meat (e.g., chicken, turkey, rabbit) Fish Dishes prepared from legumes (e.g., beans, peas, soybeans, lentils) Fruit Vegetables	Refined bread and bakery products (e.g., wheat bread, rye bread, mixed wheat–rye bread, toast bread, bread rolls, croissants) White rice, refined pasta, or finely ground cereals (e.g., semolina, couscous) Fast food (e.g., French fries, hamburgers, pizza, hot dogs, casseroles) Fried meat- or flour-based dishes Butter used as a spread or for cooking, frying, or baking Lard used as a spread or for cooking, frying, or baking Ripened cheeses (including hard and semi-hard varieties, as well as mold-ripened cheeses) Processed meat products (e.g., cold cuts, sausages, frankfurters) Dishes prepared from so-called red meat (e.g., pork, beef, veal, mutton, lamb, game) Sweets (e.g., candies, cookies, cakes, chocolate bars, muesli bars, and other confectionery products) Canned meat products Sugar-sweetened carbonated and non-carbonated beverages (e.g., Coca-Cola, Pepsi, Sprite, Fanta, lemonade) Energy drinks (e.g., Red Bull, Burn, Black Horse, 2KC, Shot, or similar products) Alcoholic beverages

**Table 2:** Scoring system for food consumption frequency categories [9].

Frequency Category	Rank Assigned to Frequency Category	Daily Frequency (Times/Day)
Never	1	0
1–3 times per month	2	0.06
Once per week	3	0.14
Several times per week	4	0.5
Once per day	5	1
Several times per day	6	2

**Table 3:** Characteristics of the study participants.

	Total (*n* = 45)	Group I (*n* = 22)	Group II (*n* = 23)	*p*	q
Age (years)	33.4 ± 3.3	33.3 ± 3.4	33.5 ± 3.3	0.837 ^	0.837
Weight (kg)	58.7 ± 7.4	53.6 ± 5.21	63.5 ± 5.9	<0.001 ^*	<0.001 *
BMI ^1^ (kg/m^2^)	21.1 ± 2.1	19.5 ± 1.3	22.6 ± 1.4	<0.001 ^*	<0.001 *
Fat mass (%)	24.9 (20.1–26.8)	20.1 (17.4–22.1)	26.8 (26.1–29.9)	<0.001 ^#^*	<0.001 *
Total body water (%)	52.3 (51.1–55.4)	55.5 (53.9–56.8)	51.1 (49.0–51.7)	<0.001 ^#^*	<0.001 *
Fat free mass (kg)	42.0 ± 3.2	40.7 ± 2.9	43.3 ± 3.1	<0.006 ^*	0.007 *
Fat free mass (%)	71.3 (69.5–75.8)	75.8 (73.9–78.1)	69.5 (66.6–70.1)	<0.001 ^#^*	<0.001 *
BMR ^2^ (kcal)	1318 (1264–1374)	1265 (1214–1317)	1359 (1310–1470)	0.001 ^#^*	<0.001 *
Abdominal circumference (cm)	78.9 ± 9.3	72.5 ± 6.3	85.0 ± 7.4	<0.001 ^*	<0.001 *
Waist circumference (cm)	69.0 (67.0–75.0)	66.5 (64.0–67.8)	75.0 (71.0–78.0)	<0.001 ^#^*	<0.001 *
Hip circumference (cm)	94.4 ± 4.7	90.9 ± 3.7	97.7 ± 2.8	<0.001 ^*	<0.001 *
WHR ^3^	0.75 (0.72–0.77)	0.74 (0.71–0.76)	0.76 (0.74–0.78)	0.010 ^#^*	0.011 *

^ *p*-values for normally distributed variables obtained by Student’s *t*-test, ^#^ *p*-values for non-normally distributed variables obtained by Mann–Whitney U test; q-values represent *p*-values adjusted for multiple comparisons using the Benjamini–Hochberg false discovery rate procedure; values are presented as mean ± SD or median (IQR) for normally or non-normally distributed variables, respectively; * statistically significant (*p* < 0.05 or q < 0.05); ^1^ BMI—body mass index, ^2^ BMR—basal metabolic rate, ^3^ WHR—Waist—Hip Ratio (waist-to-hip circumference).

**Table 4:** Energy intake and selected nutrients.

	Group I (*n* = 22)	Group II (*n* = 23)	*p*	q
Dietary energy (kcal)	1557 (1436–1945)	1711 (1511–2016)	0.254 ^#^	0.785
Protein (g)	75.2 ± 24.2	74.0 ± 15.4	0.837 ^	0.934
Protein (g/kg body weight) ^1^	1.4 (1.1–1.6)	1.1 (1–1.3)	0.059 ^#^	0.478
Protein (%)	17.7 ± 3.6	16.8 ± 2.8	0.346 ^	0.833
Animal protein (g)	45.03 ± 25.0	45.3 ± 14.0	0.961 ^	0.965
Plant protein (g)	29.5 ± 9.8	28.1 ± 5.2	0.565 ^	0.907
Animal-to-plant protein ratio	1.75 ± 1.08	1.66 ± 0.57	0.709 ^	0.934
Fat (g)	63.3 (46.1–75.4)	56.0 (46.7–67.5)	0.625 ^#^	0.907
Fat (%)	32.8 ± 6.0	30.2 ± 6.4	0.165 ^	0.597
Saturated fatty acids (g)	21.5 ± 8.0	21.4 ± 7.1	0.965 ^	0.965
Monounsaturated fatty acids (g)	25.3 (17.8–30.5)	24.2 (18.7–26.9)	0.547 ^#^	0.907
Polyunsaturated fatty acids (g)	9.4 (8.1–12.8)	9.7 (7.6–10.6)	0.601 ^#^	0.907
Carbohydrates (g)	196.4 (179.2–243.5)	216.4 (202.4–272.3)	0.082 ^#^	0.478
Carbohydrates (%)	48.6 (43.9–55.3)	54.0 (50.8–55.7)	0.159 ^#^	0.597
Sucrose (g)	29.4 ± 13.3	44.5 ± 28.6	0.028 ^*	0.475
Lactose (g)	8.8 (5.6–12.6)	5.4 (4.6–9.5)	0.271 ^#^	0.785
Glucose (g)	7.4 (5.8–9.4)	10.2 (7.8–12.8)	0.032 ^#^*	0.475
Fructose (g)	8.8 (6.6–12.9)	11.3 (9.5–16.3)	0.076 ^#^	0.478
Iron (mg)	11.5 ± 3.2	11.3 ± 2.1	0.827 ^	0.934
Calcium (mg)	763.7 (614.6–872.3)	730.9 (508.8–899.0)	0.521 ^#^	0.907
Phosphorus (mg)	1177 (1072–1469)	1228 (1078–1321)	0.779 ^#^	0.934
Magnesium (mg)	322.6 ± 97.1	313.8 ± 68.0	0.726 ^	0.833
Vitamin A (µg)	925 (762–1295)	1001 (796–1339)	0.373 ^#^	0.597
Beta-carotene (µg)	3299 (1967–4816)	4497 (2590–6550)	0.144 ^#^	0.934
Vitamin E (mg)	10.2 (8.5–12.5)	9.8 (8.5–12.2)	0.812 ^#^	0.907
Vitamin C (mg)	139.2 (86.5–188.7)	141.8 (97.9–195.9)	0.536 ^#^	0.965
Folate (µg)	367.6 ± 142.5	364.3 ± 72.5	0.923 ^	0.833
Cholesterol (mg)	246.1 (164.9–331.7)	284.0 (208.8–339.9)	0.364 ^	0.907
Dietary fiber (g)	20.9 ± 7.2	21.9 ± 5.4	0.584 ^	0.785

^1^ expressing protein intake in g/kg body weight is consistent with current nutritional recommendations and allows assessment of protein adequacy adjusted to individual body mass; ^ *p*-values for normally distributed variables obtained by Student’s *t*-test; ^#^ *p*-values for non-normally distributed variables obtained by Mann–Whitney U test; q-values represent *p*-values adjusted for multiple comparisons using the Benjamini–Hochberg false discovery rate procedure; values are presented as mean ± SD or median (IQR) for normally or non-normally distributed variables, respectively; * statistically significant (*p* < 0.05).

**Table 5:** Nutrient intake characteristics according to Polish Nutritional Standards.

Nutrient	Total/Group	Intake Below Recommendation, *n* (%)	Intake Above Recommendation, *n* (%)	Polish Nutritional Standards [10]
Protein	Total	5 (11.1)	40 (88.9)	0.9 g/kg body mass
	I	2 (9.1)	20 (90.9)	
	II	3 (13.0)	20 (87.0)	
Fat	Total	2 (4.4)	13 (28.9)	20–35% of energy
	I	1 (4.5)	10 (45.5)	
	II	1 (4.3)	2 (8.7)	
Sucrose ^1^	Total	ND	36 (80.0)	≤5% of energy
	I		17 (77.3)	
	II		19 (82.6)	
EPA + DHA	Total	33 (73.3)	ND	≥250 mg/d
	I	14 (63.3)		
	II	19 (82.6)		
SFA	Total	ND	28 (62.2)	≤10%
	I		13 (59.1)	
	II		15 (65.2)	
Dietary fiber	Total	30 (66.7)	ND	≥25 g/d
	I	14 (63.6)		
	II	16 (69.6)		
Iron	Total	45 (100)	ND	≥18 mg/d
	I	22 (100)		
	II	23 (100)		
Calcium	Total	39 (86.7)	ND	≥1000 mg/d
	I	17 (77.3)		
	II	22 (95.7)		
Magnesium	Total	25 (55.6)	ND	310 mg/day for women aged 19–30 320 mg/day for women aged 31–50
	I	11 (50.0)		
	II	14 (60.9)		
Vitamin C	Total	7 (15.6)	ND	≥75 mg/d
	I	4 (18.2)		
	II	3 (13.0)		
Vitamin E	Total	9 (20.0)	ND	≥8 mg/d
	I	4 (18.2)		
	II	5 (21.7)		
Vitamin A	Total	8 (17.8)	2 (4.4)	≥700 µg/d Toxic dose—3000 µg/d
	I	4 (18.2)	1 (4.5)	
	II	4 (17.4)	1 (4.3)	

ND—no data; ^1^ Sucrose intake was assessed based on nutrient composition data available in the dietary analysis software. Data regarding free sugars or added sugars were not available separately.

**Table 6:** Mean values of the pHDI and nHDI indices (after conversion into points) and the DQI for the entire study group and according to body fat content.

	Group I (*n* = 22)	Group II (*n* = 23)	*p*
pHDI	24.79 ± 9.43	24.20 ± 5.42	0.798 ^
nHDI	10.64 (5.72–13.39)	10.64 (7.22–15.14)	0.388 ^#^
DQI	14.55 ± 11.94	12.18 ± 8.84	0.456 ^

^ *p*-values for normally distributed variables obtained by Student’s *t*-test, ^#^ *p*-values for non-normally distributed variables obtained by Mann–Whitney U test; values are presented as mean ± SD or median (IQR) for normally or non-normally distributed variables, respectively.

## Data Availability

The raw data supporting the results and conclusions of this article are available from the corresponding author upon reasonable request.

## Supplementary Materials

The following supporting information can be downloaded at: https://www.mdpi.com/article/10.3390/nu18111775/s1; Table S1. Results of correlation analyses with Benjamini–Hochberg correction.
