# Supplementary material for: Diet Quality, Nutrient Intake, and Body Fat Percentage in Women with Infertility and Normal Body Mass Index
Source: Nutrients. 2026 May 31;18(11):1775. doi: 10.3390/nu18111775 (PMC13259199; doi:10.3390/nu18111775)
Supplement: Supplementary file 1 [file nutrients-18-01775-s001.zip › nutrients-4303435-supplementary.pdf]

Supplementary Table S1. Results of correlation analyses with Benjamini–Hochberg correction.

	r	95% CI	p	q
**Body fat (%)**				
Abdominal circumference (cm)	0.85	[0.741, 0.915]	<0.001*	<0.001*
WHR	0.539	[0.292, 0.719]	<0.001*	0.002*
pHDI	-0.01	[-0.303, 0.284]	0.947	0.969
nHDI	0.125	[-0.175, 0.404]	0.412	0.635
DQI	-0.127	[-0.405, 0.173]	0.408	0.635
Dietary energy (kcal)	0.095	[-0.205, 0.378]	0.536	0.738
Protein intake (g/kg body weight)	-0.389	[-0.613, -0.108]	0.008	0.075
Fat intake (%)	-0.211	[-0.475, 0.088]	0.165	0.343
Carbohydrates intake (%)	0.205	[-0.095, 0.470]	0.177	0.355
Sucrose intake (g)	0.335	[0.046, 0.572]	0.024*	0.142
**Protein (g/kg body weight)**				
Abdominal circumference (cm)	-0.323	[-0.563, -0.033]	0.030*	0.142
WHR	-0.184	[-0.453, 0.116]	0.226	0.407
pHDI	0.013	[-0.282, 0.305]	0.933	0.969
nHDI	0.03	[-0.266, 0.321]	0.844	0.950
DQI	0.058	[-0.239, 0.346]	0.704	0.844
Dietary energy (kcal)	0.584	[0.350, 0.749]	<0.001*	0.001*
Fat intake (%)	0.24	[-0.058, 0.498]	0.113	0.265
Carbohydrates intake (%)	-0.397	[-0.619, -0.117]	0.007*	0.075
Sucrose intake (g)	-0.009	[-0.302, 0.285]	0.951	0.969
**Sucrose (g)**				
Abdominal circumference (cm)	0.345	[0.057, 0.580]	0.020*	0.142
WHR	0.326	[0.035, 0.565]	0.029*	0.142
pHDI	-0.096	[-0.379, 0.203]	0.529	0.738
nHDI	0.298	[0.005, 0.544]	0.047*	0.158
DQI	-0.249	[-0.506, 0.048]	0.099	0.253
Dietary energy (kcal)	0.426	[0.152, 0.640]	0.004*	0.051
Fat intake (%)	-0.254	[-0.509, 0.043]	0.092	0.249
Carbohydrates intake (%)	0.317	[0.025, 0.558]	0.034*	0.142
**DQI**				
Abdominal circumference (cm)	-0.031	[-0.322, 0.265]	0.838	0.950
WHR	0.072	[-0.226, 0.358]	0.639	0.821
Dietary energy (kcal)	-0.232	[-0.492, 0.066]	0.126	0.271
Fat intake (%)	-0.001	[-0.295, 0.292]	0.993	0.993
Carbohydrates intake (%)	-0.108	[-0.389, 0.192]	0.482	0.723
Iron (mg)	0.266	[-0.030, 0.519]	0.078	0.233
Calcium (mg)	0.313	[0.021, 0.555]	0.037*	0.142
Phosphorus (mg)	0.317	[0.026, 0.559]	0.034*	0.142
Magnesium (mg)	0.278	[-0.017, 0.528]	0.064	0.205
**pHDI**				
Abdominal circumference (cm)	0.06	[-0.237, 0.348]	0.693	0.844
WHR	0.147	[-0.153, 0.423]	0.334	0.564
Dietary energy (kcal)	-0.151	[-0.426, 0.149]	0.322	0.561
Fat intake (%)	-0.01	[-0.303, 0.284]	0.948	0.969
Carbohydrates intake (%)	-0.068	[-0.354, 0.230]	0.658	0.827
Iron (mg)	0.072	[-0.226, 0.358]	0.638	0.821
Calcium (mg)	0.232	[-0.066, 0.492]	0.125	0.271
Phosphorus (mg)	0.255	[-0.041, 0.511]	0.091	0.249

Magnesium (mg)	0.136	[-0.164, 0.413]	0.372	0.608
**nHD**				
Abdominal circumference (cm)	0.049	[-0.248, 0.338]	0.749	0.879
WHR	0.095	[-0.205, 0.378]	0.537	0.738
Dietary energy (kcal)	0.322	[0.031, 0.562]	0.031*	0.142
Fat intake (%)	0.02	[-0.275, 0.312]	0.894	0.969
Carbohydrates intake (%)	0.092	[-0.207, 0.376]	0.546	0.738
Iron (mg)	-0.298	[-0.544, -0.005]	0.047*	0.158
Calcium (mg)	-0.243	[-0.501, 0.054]	0.107	0.263
Phosphorus (mg)	-0.198	[-0.464, 0.102]	0.193	0.373
Magnesium (mg)	-0.19	[-0.458, 0.110]	0.211	0.394

r – Pearson's or Spearman's correlation coefficient, depending on data distribution; 95% CI – 95% confidence interval; p – unadjusted p-value; q – p-value adjusted for multiple comparisons using the Benjamini–Hochberg false discovery rate procedure; * statistically significant ($p < 0.05$ or $q < 0.05$).
